# Modern Sociology

**Published:** 1900-01-27

**Authors:** 


### Modern Sociology.

### FRENCH FACTORY LEGISLATION.

Factory legislation in France begins, strictly speaking, with the great Revolution. In 1789, the Assembly declared the suppression of all monopolies. The intention of this was to abolish the old guild system. Henceforth, no close corporation was to be permitted to forbid the access to any art, trade, or profession to an outsider, and the decree of March, 1791, expressly provides that any one may engage in any business he may desire. But with all this liberty, there were definite limitations. Anything of the nature of a trades union was distinctly forbidden. In July of the same year, 1791, the Assembly forbade persons, whether employers or employés, to make agreements among themselves unitedly to refuse, or to accord only for certain prices, the assistance of their industry or their work. This condition continued until 1884, with the exception of a brief period after the revolution of 1848. But, as a matter of fact, after 1860 the French Government looked with a very lenient eye upon all such organisations. In 1864 the laws against coalition were formally repealed, and instead there were formulated provisions granting to both employers and employed the right to form combinations for the purpose of improving their position, but making it an offence, punishable by fine and imprisonment, to use threats or violence in the carrying out of their purpose. The fine may be anything from 16 to 3,000 francs, and the imprisonment from six days to three years; while if the offence is proved to have been committed in pursuance of a concerted plan, the period of imprisonment may be followed by a period of police surveillance of from two to five years. Trades unions may, in their corporate capacity, send in estimates and perform contracts for the central government or for the commune. One organisation is, however, exempt from these privileges. This is the famous organisation known as the International Working Men's Association. This was, as is well known, a Socialistic organisation; and it is therefore a fear of Socialism, not a dislike of trades unions, that has made the French Government object to the existence of "every international association which under any title, and especially that of the International Working Men's Association, has as its object the suspension of labour or an attack upon the right of property, the family, the country, religion, or the free exercise of religious beliefs." Affiliation with such an organisation is punished by imprisonment of from three months to two years, a fine of from 50 to 1,000 francs, and suspension from civil rights for from five to ten years. Those who are actively engaged in any propaganda connected with such an organisation may be even more severely punished.

Apprenticeship still exists in France, and seems to have a firmer hold upon the industrial classes there than in Britain. Any adult person exercising a trade can take apprentices, unless the employer has been convicted of a crime against good morals, or has been condemned to imprisonment for misdemeanour. There are some limitations, of a nature readily comprehensible, such as that which forbids an unmarried man or a widower to have female apprentices who are still in their minority lodging in his house. The duties of a master towards his apprentice are defined with the exactness which characterised mediæval laws, and are rather of a natuie that cannot well be enforced. He is to conduct himself as a good father to his young apprentice, to watch over his conduct and habits, either at home or outside, and to instruct him in his own art or trade. He is expected to inform the youth's parent or guardian of any grave offence which the apprentice may commit, or of any vicious habits he may contract. Illness must also be reported to the apprentice's friends. It is also ordained that he must not employ him to do any work that does not belong to his trade, nor may he give him a task beyond his strength or injurious to his health. It is clear that some of these conditions are of such a nature that any failure to fulfil them cannot be proved in a court of law. More practical is the law which ordains that an apprentice under the age of 14 years must not be compelled to work for more than 10 hours a day, while the working hours of those between 14 and 16 are limited to 12 hours a day, though in all conscience these hours are long enough for any young person. Still, we know that when no limitation is fixed far longer hours have been wrought by children.

The hours even of men are restricted in France to 12 per day, though the Government retains the power to designate by order those industries wherein, by reason of their nature, such a restriction is inadvisable. The Government also claims the power of allowing women to work overtime and at night in certain industries, chiefly those which can be pursued only at certain seasons, such as fruit gathering and packing, fish-curing and the like, where the object dealt with is of a perishable nature. No child under the age of 12 may be employed in a factory or workshop, and if under 13 must be able to show a satisfactory certificate of primary education. This certificate is needed even in orphan asylums and philanthropic institutions before a child can be kept at manual or technical work for more than three hours a day. Children under 13 are not permitted to be employed in theatres or music-halls, nor may any one be a member of a strolling theatrical company who has not attained the age of 16.

Women must not be employed for more than 11 hours a day, with at least one interval for rest. The total rest-time during the working day need not be more than one hour altogether, and may be given either at one time or in instalments. Most English employers would think that the energies of their workers would flag if kept at work for so long a time with no more rest than this. Men may work seven days in the week; but it is obligatory that women and children have one day's rest out of the seven. A law of 1874 fixed Sunday as the rest day; but at present any day agreed upon by employer and employed may be chosen instead. Certain legal holidays are also named when women and children are not expected to work. An exception to this rule is made in the case of establishments where it is necessary to keep up continuous fires.

As in England, the provisions of the Acts relating to the conditions of work and workers must be shown conspicuously in every workshop, and factory inspectors are appointed by the Government to see that the regulations are obeyed. The arbitration tribunal is an institution of older date in France than here. The first dates from March, 1806, and affected only the city of Lyons. This kind of court is called the "Conseil de Prud'hommes," and deals only with disputes between individuals; but of late courts have been instituted for the purpose of settling strikes and similar disputes of a larger nature.

